# Reviewer acknowledgement 2013

**DOI:** 10.1186/1756-6649-14-1

**Published:** 2014-01-17

**Authors:** Peter O'Donovan

**Affiliations:** 1 BioMed Central, 236 Gray’s Inn Road, London WC1X 8HB, UK

## Abstract

**Contributing Reviewers:**

The editors of BMC Medical Physics would like to thank all our reviewers who have contributed to the journal in Volume 13 (2013).

## 

Chamidu Atupelage

Japan

Pedro J Benito

Spain

Byungchul Cho

South Korea

Shilpee Choudhry

India

Natalia Gladkova

Russian Federation

Arto Hautala

Finland

Watchara Kasinrerk

Thailand

Zuofeng Li

USA

Romildo Nogueira

Brazil

Marc Normandin

USA

Lucia Perna

Italy

Martha Ribeiro

Brazil

Na Jin Seo

USA

Gregory Sharp

USA

Jérémy Vanhelst

France

Guobao Wang

USA

Michael Wininger

USA

## Pre-publication history

The pre-publication history for this paper can be accessed here:

http://www.biomedcentral.com/1756-6649/14/1/prepub

